# Using citizen science photographs to identify reproductive events in an oviparous elasmobranch

**DOI:** 10.1111/jfb.70044

**Published:** 2025-04-08

**Authors:** Rachel Mawer, Jane Dodd, James Thorburn, Neil M. Burns, David M. Bailey

**Affiliations:** ^1^ NatureScot Oban UK; ^2^ University of Glasgow Glasgow UK; ^3^ University of Plymouth Plymouth, UK; ^4^ Edinburgh Napier University Edinburgh UK

**Keywords:** citizen science, critical habitats, flapper skate, Rajidae, reproductive cycle

## Abstract

Identifying critical habitats is important for the effective management of vulnerable species. Critical habitats, such as mating or nursery grounds, support populations during key life stages and help to maximise reproductive output and population growth. In elasmobranchs, mating often happens over a defined season, suggesting sites associated with this process may only require temporal protection. However, knowledge gaps on such sites exist for many elasmobranchs due to the challenges associated with identifying temporal mating periods, which hinders conservation efforts. Here, we investigated the application of photographs to estimate reproductive timing in an oviparous elasmobranch, the flapper skate (*Dipturus intermedius*), as a non‐invasive and low‐cost alternative to other approaches. Using a pre‐existing citizen science photo‐ID database of over 2000 images, we identified signs of reproductive behaviour: the presence or absence of pelvic swelling, bite wounds and scratch wounds. Statistical models were created for each feature to explore seasonal trends and other parameters explaining their presence. Seasonal trends were present for all features and feature occurrence differed with sex. The occurrence of bite wounds and pelvic swelling in flapper skate peaked over winter and spring months, suggesting a winter–spring mating and egg‐laying period. These results are corroborated by previous reproductive research on the flapper skate, suggesting the applied method is a valid tool to estimate reproductive timing in an elusive elasmobranch. The approach could be applied to other flapper skate populations and other elasmobranch species, helping to close existing knowledge gaps on reproductive behaviours.

## 1 INTRODUCTION

Critical habitats are a focus of marine protected area (MPA) designation for many species, including elasmobranchs. Critical habitats are areas that support key periods in a species life history, for example mating, egg‐laying or nursery grounds. During such periods, populations experience heightened vulnerability to disturbance (McInturf et al., 2023) and ensuring sufficient protection is in place is important. In the case of the egg‐laying flapper skate (*Dipturus intermedius* Parnell, 1837), eggs will rest on the seafloor for around 18 months (Benjamins et al., 2021), thus protecting such areas is vital to maintaining population growth (Dodd et al., 2022). Historically MPAs for viviparous elasmobranchs have focused on nursery areas (Kinney & Simpfendorfer, 2009), yet such areas will largely protect juveniles and the protection offered to adults will vary. There is a need to identify critical habitat associated with other life‐history events such as mating to ensure adequate protection is in place for mature individuals to ensure all classes within the population are protected (Barnett et al., 2019; Kinney & Simpfendorfer, 2009; Pratt et al., 2022).

Mating habitats are crucial for reproductive success, increasing genetic diversity and maximising population recruitment. Mating habitats may be used by elasmobranchs in aggregations (McInturf et al., 2023) and repeatedly used by the same individuals over decades (Pratt et al., 2022). Identifying mating habitats and protecting such areas can boost long‐term spatial protection measures (Barnett et al., 2019; Chin et al., 2023), increasing the number of reproductive individuals and enhancing population recruitment (Knip et al., 2012). To date, few elasmobranch mating grounds have been definitively identified (Chapman et al., 2015; Pratt et al., 2022).

An important step towards identifying mating grounds is to determine the timing of mating events, after which spatial usage during the period can be investigated. Knowledge of mating timing is lacking for many elasmobranch species (Cailliet, 2015), which include some of the most threatened marine species (Dulvy et al., 2021). Various factors cause the data gap. Direct observation opportunities are limited for aquatic species, especially those associated with deeper water. Often studies exploring elasmobranch reproductive behaviour and timing are based in aquaria and only involve species regularly kept in captivity (Gao et al., 2022; Luer & Gilbert, 1985; Nozu et al., 2018; Parent et al., 2008). Other approaches involve the study of deceased specimens (Henderson et al., 2005; Kajiura et al., 2000) but for vulnerable and endangered species it is important to develop non‐lethal approaches (Thorburn et al., 2023). Recent non‐invasive approaches such as blood hormone analysis and ultrasounds can provide insights (Awruch, 2013; Sulikowski et al., 2004; Thorburn et al., 2023) yet require dedicated studies with specialised equipment that may result in low sample sizes relative to time and labour input. Non‐invasive and low‐cost methods could provide an additional data source for estimating elasmobranch reproductive timings in challenging species. For species frequently encountered by members of the public (e.g. via scuba diving or recreational angling), citizen science could provide a route to address data gaps.

Lack of knowledge on reproductive timing has been identified as a feature hindering the conservation of the flapper skate, a critically endangered elasmobranch and the largest skate found in European waters (Dulvy et al., 2006; Garbett et al., 2021). Flapper skate are a protected feature of the Loch Sunart to Sound of Jura (LSTSOJ) MPA on the west coast of Scotland. Within the MPA, the flapper skate population is monitored via photo‐ID, with images of skates' dorsal sides submitted by recreational anglers and the unique spot patterns used to identify individuals (Benjamins et al., 2018). While primarily a tool for identification, external signs of reproductive behaviours may also be visible in the photographs, potentially providing another avenue to identify reproductive periods. For example, biting is an integral component of elasmobranch mating, where males bite females as pre‐copulatory behaviours or to facilitate clasper insertion (Arnés‐Urgellés et al., 2018; McCallister et al., 2020; Pratt & Carrier, 2001), and resulting bite wounds can be visible in images (Garla et al., 2022; Talwar et al., 2023; Whitney et al., 2012). Photographs may also show signs of egg‐laying behaviours. External signs of egg‐carrying have been recorded in Kong skate (*Okamejei keojei* Müller & Henle, 1841) and clearnose skate (*Rostroraja eglanteria* Bosc, 1800), where egg cases caused externally visible swelling over the uteri shortly before egg‐laying (Gao et al., 2022; Luer & Gilbert, 1985). Such swelling could also be apparent in images and serves as an indicator of when skate may lay eggs. Considering this, the existing flapper skate photo‐ID database could be a valuable resource for investigating their reproductive timings.

Here, we investigate the application of photographs collected via recreational angling for photo‐ID purposes to estimate reproductive timing in the flapper skate. Specifically, we looked for marks related to mating behaviour (such as bite and scratch wounds) and for whether females carrying encapsulated eggs could be identified. Seasonal trends were analysed by fitting statistical models to investigate whether seasonal trend or prevalence of observable features differed with sex and with MPA region. If photographs could be used in this way, the method would be particularly relevant given the large number of elasmobranch photo‐ID databases for many species that exist globally.

## 2 MATERIALS AND METHODS

### 2.1 Study site and data collection

**FIGURE 1:**
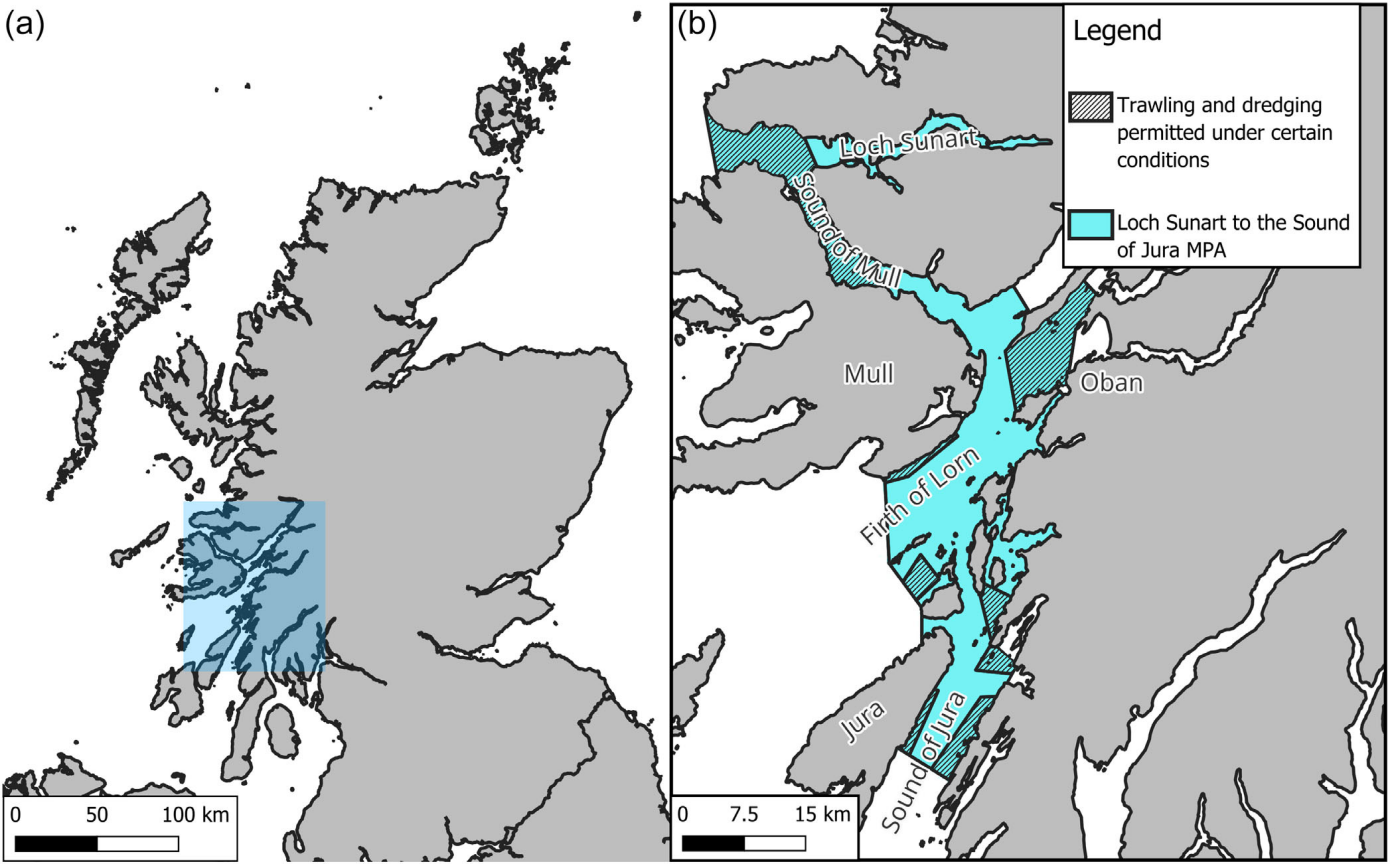
(a) Map of Scotland, with the location of the Loch Sunart to Sound of Jura marine protected area (MPA) highlighted in blue. (b) The extent of the Loch Sunart to Sound of Jura MPA shown in blue with MPA areas where trawling and dredging is permitted shaded. Regions of the MPA—Loch Sunart, the Sound of Mull, the Firth of Lorn and the Sound of Jura—are labelled. The Isles of Mull and Jura are also labelled. The town of Oban is also marked.

The study used images taken of skate captured in the LSTSOJ MPA (Figure 1). The MPA was designated in 2014 and is largely closed to fishing activities. Certain parts of the MPA are open between 1 October and 31 March, with gear modifications to reduce skate bycatch (Figure 1b). Photographs were provided via the Skatespotter project, a citizen science photo‐ID database led by NatureScot (https://skatespotter.sams.ac.uk/). Images used in this study were taken between April 2011 until the end of May 2019, comprising 1375 individual skate, many with repeat images. Images were from either the Firth of Lorn (FOL) or Sound of Jura (SOJ) regions of the MPA (Figure 1b), which host distinct populations of flapper skate (Régnier et al., 2024). Individual skate were identified as part of the ongoing photo‐ID monitoring in the MPA.

### 2.2 Ethics statement

Data used in this study came from the Skatespotter citizen science database and no new data were collected for the purpose of this study. Flapper skate anglers who contribute images to Skatespotter are referred to the Skate Handling Guide (https://skatespotter.sams.ac.uk/guides/handling.php) to encourage best practice when angling for and handling flapper skate. This guide was written by NatureScot (the Scottish Government body for conservation) in collaboration with experienced flapper skate anglers.

### 2.3 Image analysis

Skate images from the photo‐ID database were examined for three potential signs of reproductive activity: dermal wounds (bite and scratch wounds) and pelvic swelling. First, three groups of images were formed, one per feature, for further examination. Groups were formed based on image quality, to only include images where the absence of a feature could be confidently determined, that is the image showed enough of the skate dorsal surface and with sufficient resolution to conclude if a feature is not present. Image quality differed with feature as different criteria were needed, for example for bite wounds, the entire dorsal surface needed to be visible while for pelvic swelling only the pelvic region was needed (more details in Supporting Information).

A total of five observers independently examined each image. Two of the observers were co‐authors with prior expertise on flapper skate while the remaining were volunteers with no prior knowledge of skate reproduction. For all three image groups, images were examined in chronological order for each individual where multiple capture events existed. Images of male and female skate were viewed concurrently to reduce confirmation bias. No references to where skate were captured in the MPA were provided to observers. The date was embedded in the file name as part of the filing convention used for MPA monitoring and due to the quantity of data it was not possible to remove such information.

Presence or absence of pelvic swelling, bites and scratches were recorded according to predetermined criteria supplied to observers (Table 1 and Supporting Information). For bite wounds where an individual skate had multiple images, observers recorded whether a new bite was present compared to the last available image for that individual. This procedure was included due to recaptures occurring over relatively short time frames and to account for the same wound being recorded in multiple images. For further analysis, only the “new” recording of a bite was used: this was to ensure the first occurrence of a bite in the database was used in analysis. For pelvic swelling, a third category of “undetermined” was also assigned for cases where the criteria for presence or absence were not met.

**TABLE 1:** Criteria used to score images on the presence or absence of pelvic swelling, bite wounds and scratch wounds.

Feature	Scoring	Verbal description
Pelvic swelling	Yes	i. Swollen appearance, particularly in latter half of pelvic disc, giving pelvic disc an inflated appearance
		ii. Lack of definition at edges of back‐bone
	No	i. Pronounced depression in central disc
		ii. No swelling or bulging
	Undetermined	i. Lack of major swelling coupled with no major depression
		ii. Back‐bone defined along the edge without general pronounced depression
Bite wounds	Yes	i. U‐shaped collection of equidistant marks, generally around edge of body
		ii. Triangular arrangement of scratch equidistant marks on wings or pelvic disc
		iii. Single‐track or double‐track equidistant scrapes on body surface
		Separately record bites which are new to the observer and general presence, e.g. record new bites and overall presence
	No	None of the above are applicable
Scratches	Yes	i. Marks running approximately parallel to the anterior–posterior axis across the pectoral fin(s)
		ii. Marks are not a clear, distinct, constant line
		iii. Scratch(es) not visible in previous image of individual (if applicable/possible to determine)
	No	None of the above are applicable

*Note*: For the version including examples, see Supporting Information section “Criteria for scoring images”.

The majority scoring for an image was used for statistical analysis (i.e. minimum three out of five observers agreed). If no clear majority existed for pelvic swelling analysis, the image was assigned “undetermined”. All “undetermined” images were removed from further analysis. After scoring, datasets were randomly subsampled to retain only one entry per individual due to a wide variation in the number of individual recaptures. Most individuals (around two‐thirds) had only one image in each data pool while approximately 5% had over five.

### 2.4 Statistical analysis

All statistical analyses and visualisation were conducted using R version 4.4.2 (R Core Team, 2024). For pelvic swelling, a preliminary model was fitted. As one hypothesis was that the pelvic swelling was indicative of fully formed egg cases, an initial generalised linear model (GLM) with a binomial error structure (Fieberg, 2024) was made to determine if pelvic swelling occurrence differed significantly between the sexes (formula: swelling ~ sex).

For all datasets, generalised additive mixed models (GAMMs) with a binomial error structure were created using the mgcv package (Wood, 2017) to model parameters affecting the presence or absence of a feature. Backwards stepwise model selection was used to find the most parsimonious model using Akaike's information criterion (AIC) to compare models (Sutherland et al., 2023). Model selection started from a saturated model, which included sex, region of the MPA and an interaction between sex and region as fixed effects. Day of the year was fitted as a cyclic cubic regression spine. In the saturated models, separate splines were fitted per sex and per region to test whether seasonal differences occurred between either group. Skipper and year were included as random effects. With pelvic swelling, if male skate exhibited no seasonal patterns in swelling occurrence while females did in the saturated model, the data was filtered to include only female skate and the female‐only data used in model selection. Terms were removed step‐by‐step from the saturated model, with the intention of minimising model AIC. A more complex model was only retained if it reduced the AIC by more than 2 units.

## 3 RESULTS

### 3.1 Overview of images

**FIGURE 2:**
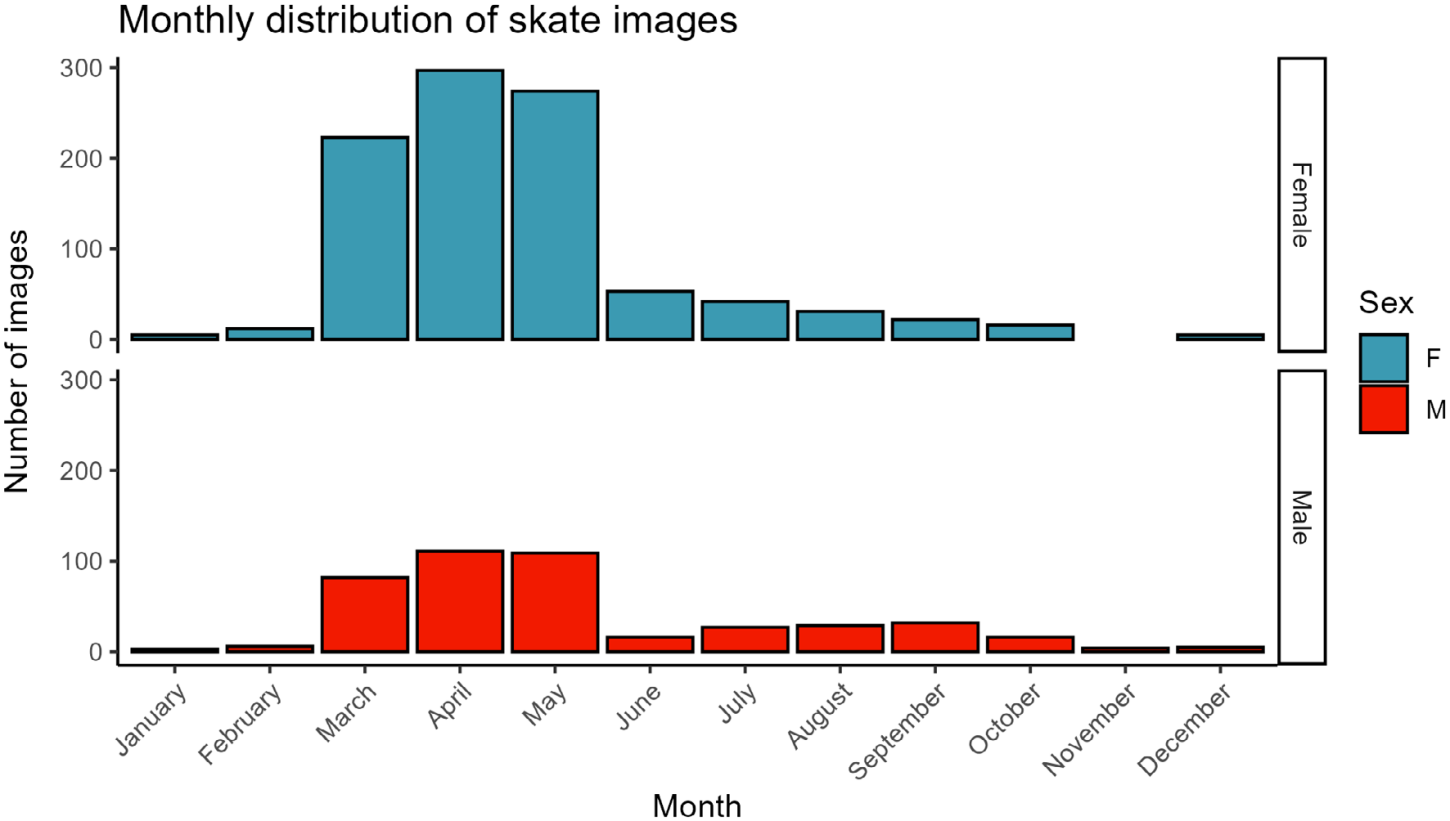
Number of images included in the statistical analysis per sex per month across all features. If an image was used in analysis for more than one feature, it was only counted here once. F, female; M, male.

Total images analysed were 2307 for pelvic swelling, 1266 for bite wounds and 1755 for scratch wounds. A difference in total images emerged as bite wounds required images showing the entire skate dorsal surface in sufficient resolution with minimal glare/shadows. After subsampling to retain one image per individual, 1420 unique images were present across the three features. The numbers of images used in the statistical analysis were 996, 716 and 993 for pelvic swelling, bite wounds and scratch wounds, respectively. Final counts of total images per month used in statistical analyses are shown in Figure 2. Images were not available uniformly throughout the year, with most images taken in the spring months. Of the images in the statistical analyses, across all features, 828 were from the FOL and 592 from the SOJ. Female skate accounted for 980 images while 440 images were for males.

### 3.2 Reproductive features visible in images

**FIGURE 3:**
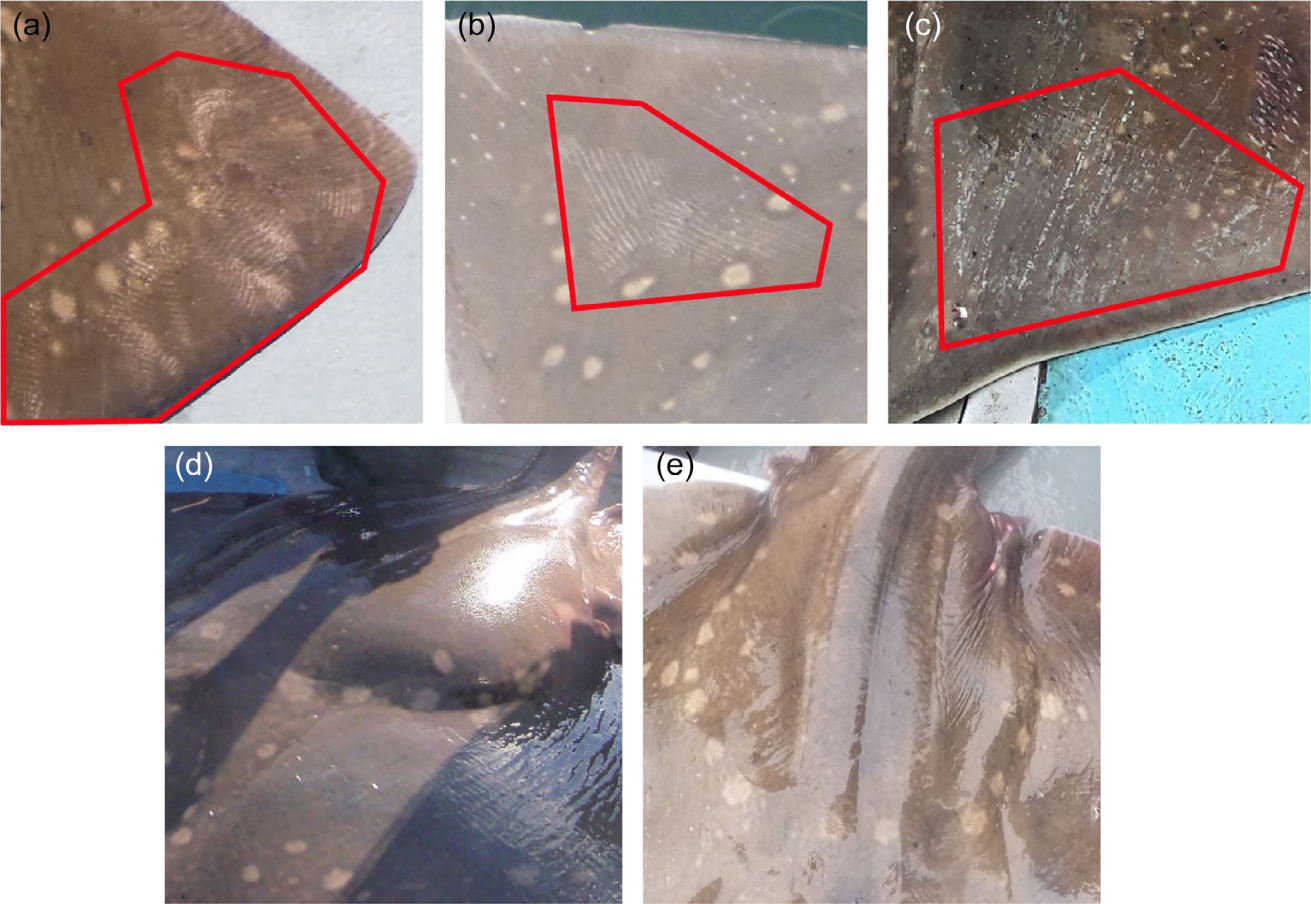
Examples of (a) and (b) bite wounds, (c) scratch wounds and (d) pelvic swelling with (e) showing the appearance when there is no pelvic swelling. The red polygons in (a)–(c) enclose the bites and scratches.

Three potential features of reproductive behaviour were visible in flapper skate images: (1) bite wounds, as a sign of mating (Figure 3a,b); (2) scratch wounds, as a sign of potential competition between males during mating (Figure 3c); and (3) pelvic swelling, as a sign of egg cases and indicator of egg‐laying behaviour (Figure 3d).

### 3.3 Seasonal trends in feature occurrence

Final models are shown in Table 2 and model selection is summarised in Table S1.

**TABLE 2:** Final generalised additive mixed models for pelvic swelling, bite wound and scratch wounds in flapper skate images.

Feature	Fixed effects retained in final model
Pelvic swelling (female only)	Presence~ s(day, bs = ‘cc’)
Bite wounds	Presence~ sex +s(day, bs = ‘cc’)
Scratch wounds	Presence~ sex +s(day, bs = ‘cc’)

*Note*: For pelvic swelling, model selection was only applied to female skate as there was no seasonal variation in swelling for male skate.

#### 3.3.1 Bite wounds

**FIGURE 4:**
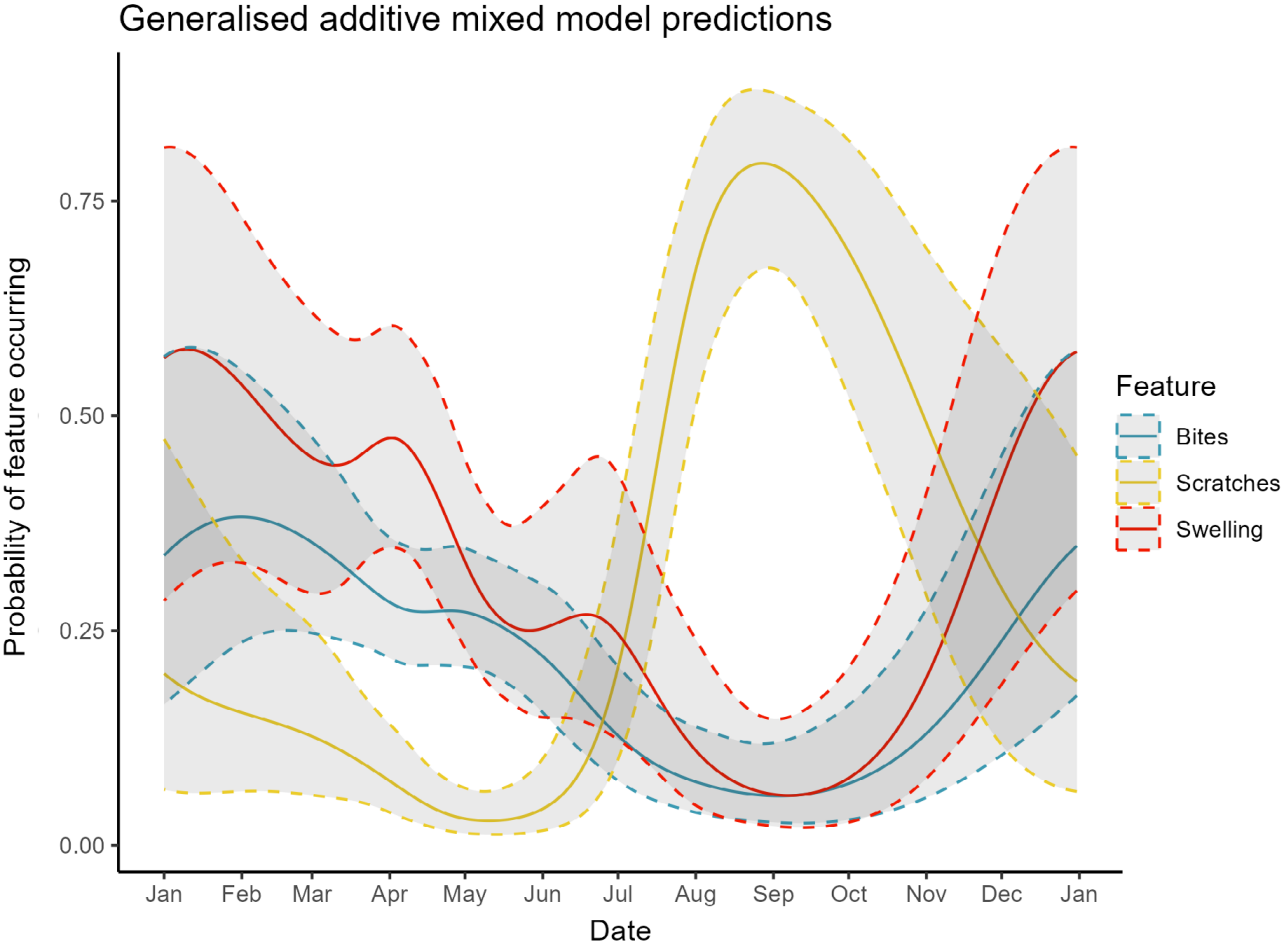
Predicted probability of occurrence of bite wounds, scratches and swelling in images of skate, from the separate final models made for each feature. Dashed lines indicate the 95% confidence intervals for the fixed‐effects predicted probabilities. Model predictions are shown for female skate (swelling and bites) or males (scratches) in the Firth of Lorn, as there was no seasonal difference between regions. Note that for swelling, only female skate were modelled. There were no interactions between date and other parameters. For predictions, the year was set to 2019 and the skipper to “RC”.

**FIGURE 5:**
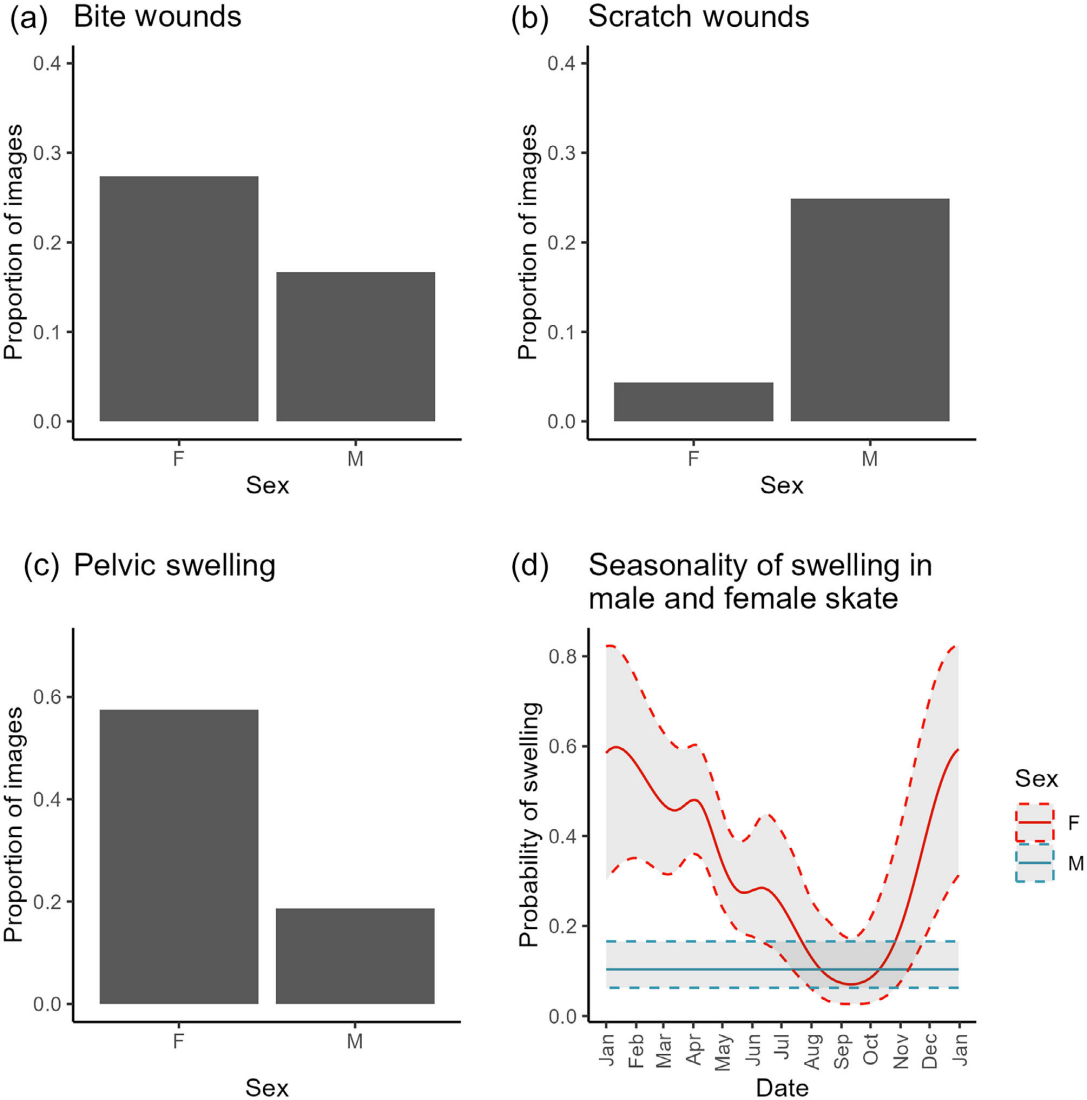
Sex‐specific differences in the occurrence of pelvic swelling, bite wounds and scratch wounds in flapper skate images. Proportion of images showing (a) bite wounds, (b) scratch wounds and (c) pelvic swelling, per sex. (d) General additive mixed model predictions for the seasonal probability of pelvic swelling in male and female skate, showing 95% confidence intervals. F, female; M, male.

**FIGURE 6:**
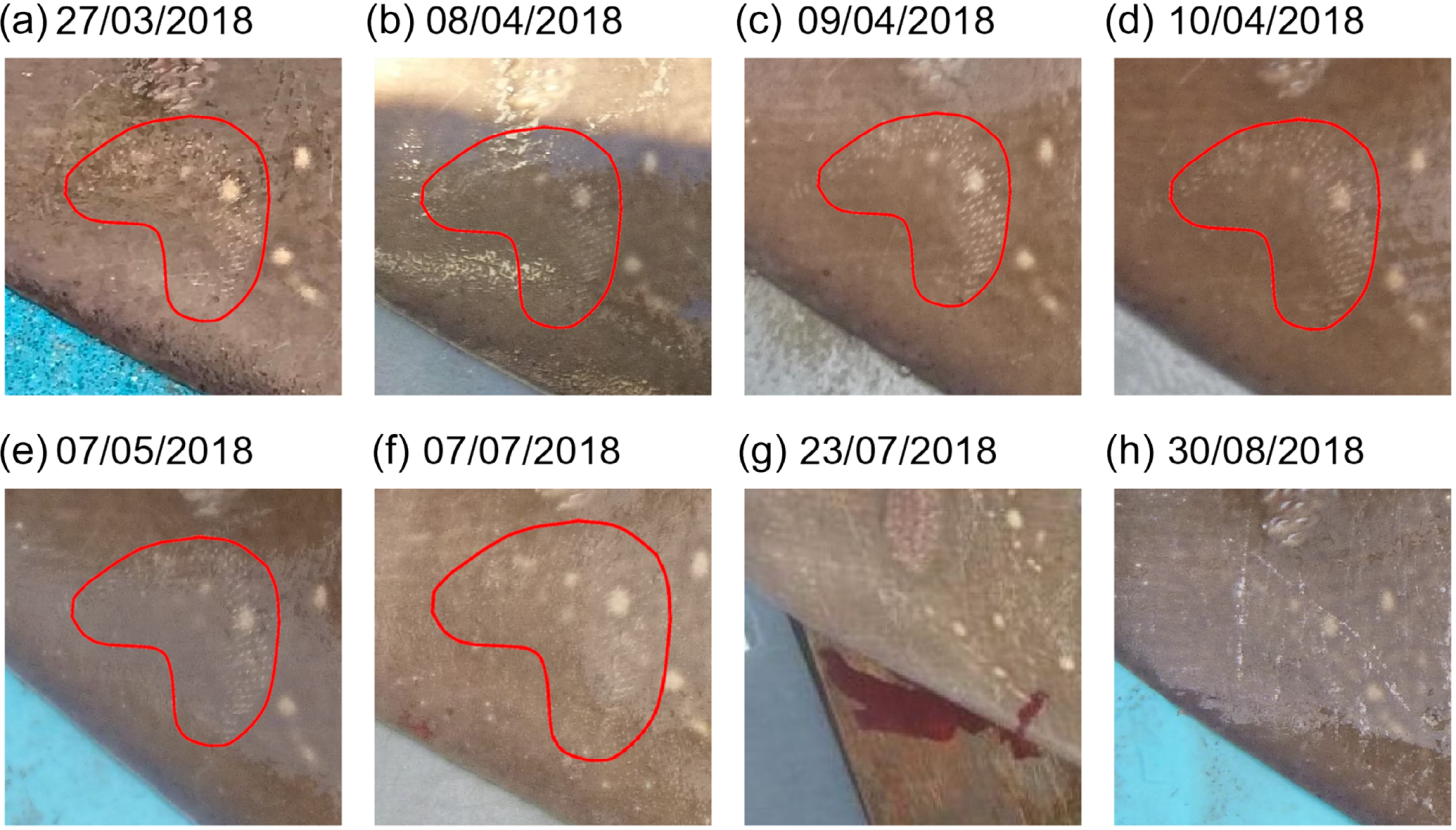
Repeated occurrence of a bite wound on one skate individual, shown in the red circle, for recaptures spanning 102 days, and two subsequent recaptures where the specific wound is no longer visible. The red shape is drawn around the specific bite wound. Note that not all images were included after filtering images on quality prior to scoring by the five observers. All images were taken in 2018 on (a) 27 March, (b) 8 April, (c) 9 April, (d) 10 April, (e) 7 May, (f) 7 July, (g) 23 July and (h) 30 August.

Bite wound occurrence varied throughout the year (Figure 4 and Table 1). Probability of a bite being present in an image was highest from November until May and lowest around September. While there were no seasonal differences in bite occurrence between males and females, overall females were more likely to have bite wounds: 27.37% of images of female skate featured a new bite wound compared to 16.67% of male images (Figure 5a). Photographs also provided insight into the persistence of individual bite wounds on skate. For example, after initial scoring, it was noticed that the same bite wound was observable on one individual for multiple recaptures over a long period. On further investigation looking at all available images, the wound was visible across 102 days, between 27 March and 7 July (Figure 6a–f). The wound appears, though faint, on 7 July (Figure 6f). The same skate was recaptured again after 7 July, with the bite no longer clear on 23 July (where the angle is not perfect for this specific wound if faded) or 30 August, 118 and 156 days, respectively, after the first observation (Figure 6g,h).

#### 3.3.2 Scratch wounds

Scratch wound occurrence was explained by sex and varied seasonally (Table 2 and Figure 4). Male skate were more likely than females to feature scratch wounds (24.8% of male images had scratches compared to 4.4% of female; Figure 5b). Both sexes had the same seasonal trend in scratch occurrence, which peaked around September and was at its lowest in May (Figure 4).

#### 3.3.3 Pelvic swelling

The initial GLM to compare if there were a significant difference in the probability of swelling in female vs. male skate suggested there was a significant difference (*P* < 0.001). The proportion of images of female skate with swelling was approximately three times that of males (Figure 5c). Moreover, the initial GAMM including both sexes in the data and with an interaction between sex and date showed a seasonal trend for female skate but not males (Figure 5d). As a result, further model selection proceeded using only data on female skate.

For the final model for female skate, pelvic swelling was explained by date. Probability of pelvic swelling was highest from November to early May and lowest in September (Figure 4).

## 4 DISCUSSION

In this study, an existing photograph database for a critically endangered elasmobranch was used to investigate reproductive timing in a non‐invasive manner. The investigated features—pelvic swelling, bite wounds and scratch wounds—showed seasonal patterns. Both the seasonal patterns along with literature on other skate and elasmobranch species suggest the features represent behaviours linked to reproduction. The results can be interpreted to suggest when flapper skate mate and deposit eggs within the study site.

Bite wounds were more likely to occur between November and May. Biting is a common behaviour across elasmobranchs during mating (Arnés‐Urgellés et al., 2018; McCallister et al., 2020). While bites can be produced by other behaviours (Domeier & Nasby‐Lucas, 2007), bite wounds are often most prevalent during known mating periods in other elasmobranchs and occur more on females (Kajiura et al., 2000; Pratt & Carrier, 2001). As a result, the November to May trend for bite wounds suggests that flapper skate have a discrete mating period from winter into spring.

The scratch wounds showed the opposite trend. Scratch wounds had a clear peak in September and occurrence was at low levels over winter. Prior to analysis, scratch wounds were hypothesised as another product of mating‐related behaviours, for example aggression between males in competition for females (Shibuya & Duncan, 2022), but results suggest that is not the case. Given the strong seasonal trend for scratch wounds along with the greater occurrence on male skate, its occurrence may not be random and may be due to another aspect of as‐yet unknown skate behaviour. For example, scratch wounds could result from male–male competition prior to mating, for example for food or space. Alternatively, it could be due to other behaviours discrete from reproduction, for example other social interactions or environmental factors. While the precise cause of scratch wounds is unknown, we suggest it may be due to the sharp claspers being dragged over the disc or raking of the lower jaws over the disk. Literature on other skate has no mention of similar wounds, although specific terminology may vary. Authors have observed the scratch wounds on flapper skate populations elsewhere in Scotland, suggesting the phenomena may be more widely spread and is not restricted to the MPA. Ultimately, there is great uncertainty over the cause of the scratch wounds, which limits its discussion in the context of reproduction. Further research on flapper skate behaviours (e.g. with underwater camera systems) may shed light on the cause behind scratch wounds.

Pelvic swelling had a seasonal trend only for female skate with a similar pattern to bite wounds, peaking between November and May. While some male skate were classed as “swollen”, swelling had no seasonal trend in males, indicating that there is something unique to female skate that causes both an increased incidence of pelvic swelling along with seasonal patterns. While this could be due to other behaviours such as changes in feeding patterns, given the evidence linking pelvic swelling to the presence of eggs (Gao et al., 2022; Luer & Gilbert, 1985) we assume the seasonal patterns are indicative of female skate carrying large encapsulated eggs. Recent ultrasound research (Thorburn et al., 2023) suggests that flapper skate undergo an overwinter egg‐laying period. In April 2019, a flapper skate deposited an egg on capture, which was successfully incubated and hatched in September 2020 (Benjamins et al., 2021). The timing of these events aligns with our photograph analysis, supporting a winter–spring egg‐laying period.

Together, the seasonal trends of bite wounds and pelvic swelling suggest flapper skate mate in winter to spring, with egg formation and deposition following immediately. Of note is that the trends indicate mating and egg‐laying overlap temporally. Specific mating and egg‐laying timing differ with skate species. For example, in Kong skate mating and egg‐laying periods were distinct, with egg‐laying occurring months after mating (Gao et al., 2022). By comparison, clearnose skate mating and egg‐laying periods overlap (Luer & Gilbert, 1985). While information on mating timing is lacking across *Dipturus* species, for some *Dipturus* species mating and egg‐laying is not seasonally limited while others have an overwinter period, similar to our findings for flapper skate. In the longnose skate (*Dipturus oxyrinchus* L., 1758), reproductively active adults were apparent throughout the year with peaks in occurrence, with reproductively active males mostly observed in summer and egg‐bearing females in autumn and winter (Bellodi et al., 2017). With captive barndoor skate (*D. laevis* Mitchill, 1818), egg‐laying was observed throughout the year with an autumn peak in activity, although mating itself was never observed (Parent et al., 2008). For other *Dipturus* species, overwinter reproductive periods have been described (Kyne et al., 2008; Licandeo et al., 2007). Given that skate and other elasmobranchs can store viable sperm for months, flapper skate could continue to lay eggs months after mating (Bellodi et al., 2017; Luer et al., 2007). Our proposed flapper skate mating and egg‐laying periods from the photograph analysis agree with the available literature (Benjamins et al., 2021; Thorburn et al., 2023), thereby suggesting the described approach as a valid method for estimating skate reproductive timing. The high prevalence of swollen females over winter and spring along with bite wounds suggests the MPA is of importance for flapper skate reproduction (Hyde et al., 2022).

By identifying when flapper skate may be mating, research can now be focused onto identifying where mating is occurring. Although the MPA is largely protected, in some areas seasonal demersal trawling and dredging is permitted from 1 October to 31 March, with gear modifications to limit skate bycatch (Kynoch et al., 2015). While skate movement has been studied in the MPA, flapper skate spatial usage in the winter months is unclear. During winter, female flapper skate in the MPA make greater use of shallower waters (Thorburn et al., 2021) which aligns with depths of known egg‐laying areas elsewhere (Dodd et al., 2022) yet the specific area of the MPA that they are occupying at this time is unknown. Given the potential overlap between skate mating and the seasonal trawling and dredging, there is a need to identify where skate are during this time and whether conflict exists with bottom‐contact mobile gear fishing.

While we believe photographs are a valuable tool for studying elasmobranch reproduction, there are some caveats to the approach. Using wounds as an indicator assumes wounds occurred near the time the photograph was taken. In reality, wounds may have occurred an indeterminate amount of time prior to being photographed. Wound healing times can vary across elasmobranchs. For example, minor injuries on whale sharks (*Rhincodon typus* Smith, 1828) were 80% closed within 25 days (Womersley et al., 2021), while for blacktip reef sharks (*Carcharhinus melanopterus* Quoy & Gaimard, 1824) a major bite wound was observed to be completely healed within 40 days (Chin et al., 2015). In our study we observed a long time‐to‐healing for the bite wounds, with one wound observed 102 days after its first sighting and no longer visible 118 days after its first sighting. In a previous study on bite wound prevalence in images of whitetip reef sharks (*Triaenodon obsesus* Rüppell, 1837), no seasonal trends could be observed for bite wounds as a result of indeterminate wound age (Whitney et al., 2012). While we could extract a seasonal trend in our study, it is possible that mating ends sooner than suggested but data availability prevents observing that trend. In other words, new bites may occur more over winter but are not observed until the spring months when more angling takes place, overestimating the probability of bites occurring in those months. Overall, data were lacking for winter months when we suspect mating occurs and while we could still observe potential trends, we acknowledge the associated issues. By comparison, for a skate to be visibly swollen due to the presence of eggs it is likely the skate will lay the eggs in the subsequent days and therefore swelling is more closely linked to actual deposition time. However, classing a skate as swollen or not is ultimately subjective. Our criteria, multiple observers, and the option to class a skate as “undetermined” attempted to account for subjectivity to some extent, but there is still uncertainty over how swollen a skate carrying eggs would appear to be. A dedicated study with ultrasound imaging and photographs of skate, with and without egg cases, could help with future standardisation of classification by providing definite examples of what a flapper skate looks like externally when carrying egg cases. A more rigorous scoring criteria could be developed, providing observers with clearer guidance on classifying pelvic swelling. Lastly, in this study we used a large database. It may be challenging to acquire seasonal trends from much smaller sample sizes due to caveats relating to wound age or subjectivity, especially if images throughout the year are unavailable. Ultimately, photographs provide an alternative tool for reproduction estimation and are of particular value in situations where other approaches, suchas blood sampling and ultrasound, are challenging.

Citizen science played a pivotal role in this research. The research would not have been possible without anglers' contributions, which formed the photo‐ID database, while volunteer analysis of the photographs strengthened the results of image scoring. Across ecology, the incorporation of citizen science can provide valuable, and perhaps otherwise challenging to obtain, data as well as cover key conservation gaps and increase the number of observations beyond what may otherwise be possible (Kobori et al., 2016; McKinley et al., 2017; Van Vliet & Moore, 2016). For example, citizen science data has been used to evaluate the distribution and diversity of sharks (Blanco‐Parra et al., 2022; Séguigne et al., 2023), provide direct observations of elasmobranch mating behaviours (Garla et al., 2022) and provide vast photo‐ID databases like Skatespotter, as seen in this paper. The role of citizen scientists within studies can vary, such as contributing data (Blanco‐Parra et al., 2022; Garla et al., 2022; Séguigne et al., 2023; Whitney et al., 2012) and data analysis, such as identifying species in videos and images (Green et al., 2023; Van Vliet & Moore, 2016). Anglers have contributed to flapper skate conservation in Argyll since the 1970s, when they began tagging skate in the area and the designation of the LSTSOJ MPA was based on tag/recapture data curated by Glasgow Museum and the Scottish Sea Angling Conservation Network (Sutcliffe, 1994). Anglers' continuing voluntary contributions reduces costs and enables the ongoing monitoring of skate in the MPA while their own observations have also guided research. For example, anglers have long speculated on the pelvic swelling and historical mark‐recapture data from the Scottish Shark Tagging Project contains references to “swollen” skate, linking it to gravidity as far back as the 1990s. Moreover, scratch wounds were investigated as a result of angler comments: anglers had reported the scratch wounds and thought they occurred more on male skates, speculating a link to mating. Our results agree with their speculation on the swelling and egg‐carrying, along with the increased prevalence of scratches on males, however, they suggest the scratch wounds are not mating related. Feedback to the anglers will help to continue to engage them in the project and encourage future observations that could lead to further avenues of research, continuing the useful exchange of information. Citizen science not only made the work possible and reduced costs, but adds value, engaging the public in the restoration of a critically endangered species.

Looking to the future, the presented methods can be applied to other skate populations and potentially adapted for other elasmobranchs. With lack of knowledge hampering flapper skate conservation (Garbett et al., 2021), it is imperative that more flapper skate populations are studied. While we showed two distinct flapper skate populations (Régnier et al., 2024) with identical seasonal trends, the studied skate were from within a limited geographic area. In other *Dipturus* species, reproductive traits can vary geographically (Licandeo & Cerna, 2007) and there is a need to determine whether flapper skate mating periods vary between different populations across a wider range. With flapper skate a popular target for recreational anglers across their range, it is possible to develop flapper skate photo‐ID databases using angler‐contributions elsewhere, providing an additional avenue for collecting data on reproductive timing at little cost to researchers. The methods can likely be applied to other skate species, given that both bite wounds and pelvic swelling will occur (Gao et al., 2022; Luer & Gilbert, 1985). However, for future applications to flapper skate and other skate species, there is a need for a large number of images, which could prove challenging. In the LSTSOJ MPA, dedicated flapper skate charter boats operate and provide the bulk of the images in Skatespotter, with anglers travelling from all over the world to the MPA to fish for flapper skate. Getting a similar quantity of images from other areas, without dedicated skate charters, may be difficult. As a result, while the methods presented in this paper could be applied to other flapper skate populations and skate species, acquiring enough images may be a limiting factor to their application. For other elasmobranchs, be it oviparous sharks or live‐bearing rays and sharks, some adaptions may be required to account for different body types and/or birth mode when considering pregnancy. In previous use of photographs for studying reproductive timing, girth and abdominal distension were used as signs of pregnancy (Marshall & Bennett, 2010; Whitney et al., 2012). By comparison, bite wounds are universal in elasmobranchs and a reliable indicator of reproductive activity, thus could be looked at across the taxa. Photo‐ID databases exist for many elasmobranchs (Marshall & Pierce, 2012), providing a potential wealth of data that could be analysed for identifying elasmobranch reproductive timing. Alternatively, researchers themselves could take photographs of elasmobranchs as a quick way to collect data for studying reproductive timing while conducting other research. Where photographs are taken for the specific purpose of reproductive studies, additional photographs could provide further benefit. In other elasmobranchs, the clasper glands are enlarged during mating periods (Anaya‐López & Ramírez‐Pinilla, 2017; Piercy et al., 2006) thereby potentially providing another visual metric to gauge breeding periods. Such images were not possible for the presented paper, given the specification of dorsal images for photo‐ID purposes.

The lack of data on elasmobranch reproductive timing can hinder conservation planning. Here, we made opportunistic use of an existing photo‐ID database and demonstrated that visible indicators of reproductive activity in photographs of flapper skate can be used to estimate reproductive timings in a non‐invasive manner. The presence of bite wounds, scratch wounds and pelvic swelling varied seasonally for the populations of skate within the MPA, indicating a winter and spring mating and egg‐laying period. The presented method could be replicated in other flapper skate populations and elasmobranch species, providing another tool for estimating reproductive timing in elasmobranchs.

## AUTHOR CONTRIBUTIONS

R.M., J.D. and J.T. conceptualised the study. R.M. coordinated volunteers for examining photographs. R.M. and N.M.B. analysed the data. All authors contributed to interpreting results. R.M. wrote the first draft of the paper and all authors contributed to subsequent revisions.

## Supporting information


**Data S1.** Supporting Information.

## References

[jfb70044-bib-0001] Anaya‐López, P. , & Ramírez‐Pinilla, M. P. (2017). Clasper gland morphology and development in *Potamotrygon magdalenae* (Elasmobranchii: Potamotrygonidae). Journal of Morphology, 278, 369–379. 28112880 10.1002/jmor.20647

[jfb70044-bib-0002] Arnés‐Urgellés, C. , Hoyos‐Padilla, E. M. , Pochet, F. , & Salinas‐de‐León, P. (2018). First observation on the mating behaviour of the marbled ray, *Taeniurops meyeni*, in the tropical eastern Pacific. Environmental Biology of Fishes, 101, 1693–1699.

[jfb70044-bib-0003] Awruch, C. A. (2013). Reproductive endocrinology in chondrichthyans: The present and the future. General and Comparative Endocrinology, 192, 60–70. 23763870 10.1016/j.ygcen.2013.05.021

[jfb70044-bib-0004] Barnett, A. , McAllister, J. D. , Semmens, J. , Abrantes, K. , Sheaves, M. , & Awruch, C. (2019). Identification of essential habitats: Including chimaeras into current shark protected areas. Aquatic Conservation: Marine and Freshwater Ecosystems, 29, 865–880.

[jfb70044-bib-0005] Bellodi, A. , Porcu, C. , Cannas, R. , Cau, A. , Marongiu, M. F. , Mulas, A. , Vittori, S. , & Follesa, M. C. (2017). Life‐history traits of the long‐nosed skate *Dipturus oxyrinchus* . Journal of Fish Biology, 90, 867–888. 27873321 10.1111/jfb.13205

[jfb70044-bib-0006] Benjamins, S. , Dodd, J. , Cole, G. , Naylor, A. , & Thorburn, J. A. (2021). First confirmed complete incubation of a flapper skate (*Dipturus intermedius*) egg in captivity. Journal of Fish Biology, 99(3), 1150–1154. 10.1111/jfb.14816 34076277 PMC8518643

[jfb70044-bib-0007] Benjamins, S. , Fox, C. J. , Last, K. , & McCarty, C. E. (2018). Individual identification of flapper skate *Dipturus intermedius* using a baited camera lander. Endangered Species Research, 37, 37–44.

[jfb70044-bib-0008] Blanco‐Parra, M. D. P. , Gasca, A. A. , Rincón, C. A. R. , Martínez, N. H. G. , & Niño‐Torres, C. A. (2022). Citizen science as a tool to get baseline ecological and biological data on sharks and rays in a data‐poor region. Sustainability, 14, 1–10.

[jfb70044-bib-0009] Cailliet, G. M. (2015). Perspectives on elasmobranch life‐history studies: A focus on age validation and relevance to fishery management. Journal of Fish Biology, 87, 1271–1292. 26709208 10.1111/jfb.12829

[jfb70044-bib-0010] Chapman, D. D. , Feldheim, K. A. , Papastamatiou, Y. P. , & Hueter, R. E. (2015). There and Back again: A review of residency and return migrations in sharks, with implications for population structure and management. Annual Review of Marine Science, 7, 547–570. 10.1146/annurev-marine-010814-015730 25251267

[jfb70044-bib-0011] Chin, A. , Molloy, F. J. , Cameron, D. , Day, J. C. , Cramp, J. , Gerhardt, K. L. , Heupel, M. R. , Read, M. , & Simpfendorfer, C. A. (2023). Conceptual frameworks and key questions for assessing the contribution of marine protected areas to shark and ray conservation. Conservation Biology, 37, 1–13. 10.1111/cobi.13917 PMC10107163 35435294

[jfb70044-bib-0012] Chin, A. , Mourier, J. , & Rummer, J. L. (2015). Blacktip reef sharks (*Carcharhinus melanopterus*) show high capacity for wound healing and recovery following injury. Conservation Physiology, 3, 1–9. 10.1093/conphys/cov062 PMC4778477 27293741

[jfb70044-bib-0013] Dodd, J. , Baxter, J. M. , Donnan, D. W. , James, B. D. , Lavender, E. , Mcsorley, C. A. , Mogg, A. O. M. , & Thorburn, J. A. (2022). First report of an egg nursery for the critically endangered flapper skate *Dipturus intermedius* (Rajiformes: Rajidae). Aquatic Conservation: Marine and Freshwater Ecosystems, 32, 1647–1659.

[jfb70044-bib-0014] Domeier, M. L. , & Nasby‐Lucas, N. (2007). Annual re‐sightings of photographically identified white sharks (*Carcharodon carcharias*) at an eastern Pacific aggregation site (Guadalupe Island, Mexico). Marine Biology, 150, 977–984.

[jfb70044-bib-0015] Dulvy, N. K. , Di Notarbartolo Sciara, G. , Serena, F. , Tinti, F. , Ungara, N. , Mancusi, C. , & Ellis, J. (2006). Dipturus batis (Common Skate). https://www.iucnredlist.org/species/39397/10198950 (accessed 27 August 2019).

[jfb70044-bib-0016] Dulvy, N. K. , Pacoureau, N. , Rigby, C. L. , Pollom, R. A. , Jabado, R. W. , Ebert, D. A. , Finucci, B. , Pollock, C. M. , Cheok, J. , Derrick, D. H. , Herman, K. B. , Sherman, C. S. , VanderWright, W. J. , Lawson, J. M. , Walls, R. H. L. , Carlson, J. K. , Charvet, P. , Bineesh, K. K. , Fernando, D. , … Simpfendorfer, C. A. (2021). Overfishing drives over one‐third of all sharks and rays toward a global extinction crisis. Current Biology, 31, 4773–4787.e8. 34492229 10.1016/j.cub.2021.08.062

[jfb70044-bib-0017] Fieberg, J. (2024). Statistics for ecologists: A frequentist and Bayesian treatment of modern regression models. University of Minnesota Libraries Publishing.

[jfb70044-bib-0018] Gao, G. , Xiao, Z. , Ji, G. , Xiao, Y. , Li, J. , Science, M. , Xiao, Z. , Li, J. , Program, D. , & Science, M. (2022). First observation of the mating, egg‐laying, and hatching behavior of a captive female Kong skate, *Okamejei kenojei* . Journal of Fish Biology, 101(4), 1084–1091. 35833517 10.1111/jfb.15165

[jfb70044-bib-0019] Garbett, A. , Phillips, N. D. , Houghton, J. D. R. , Prodöhl, P. , Thorburn, J. , Loca, S. L. , Eagling, L. E. , Hannon, G. , Wise, D. , Pothanikat, L. , Gordon, C. , Clarke, M. , Williams, P. , Hunter, R. , McShane, R. , Brader, A. , Dodd, J. , McGonigle, C. , McIlvenny, H. , … Collins, P. C. (2021). The critically endangered flapper skate (*Dipturus intermedius*): Recommendations from the first flapper skate working group meeting. Marine Policy, 124, 1–5.

[jfb70044-bib-0020] Garla, R. C. , Veras, L. B. , & Garrone‐Neto, D. (2022). Mating behavior of the lemon shark, *Negaprion brevirostris* (Carcharhiniformes: Carcharhinidae), as revealed by citizen science in the equatorial Atlantic Ocean. Revista de Biología Tropical, 70, 702–712.

[jfb70044-bib-0021] Green, S. E. , Stephens, P. A. , Whittingham, M. J. , & Hill, R. A. (2023). Camera trapping with photos and videos: Implications for ecology and citizen science. Remote Sensing in Ecology and Conservation, 9, 268–283.

[jfb70044-bib-0022] Henderson, A. C. , Arkhipkin, A. I. , & Chtcherbich, J. N. (2005). Distribution, growth and reproduction of the white‐spotted skate *Bathyraja albomaculata* (Norman, 1937) around The Falkland Islands. Journal of Northwest Atlantic Fishery Science, 35, 79–87.

[jfb70044-bib-0023] Hyde, C. A. , di Notarbartolo Sciara, G. , Sorrentino, L. , Boyd, C. , Finucci, B. , Fowler, S. L. , Kyne, P. M. , Leurs, G. , Simpfendorfer, C. A. , Tetley, M. J. , Womersley, F. , & Jabado, R. W. (2022). Putting sharks on the map: A global standard for improving shark area‐based conservation. Frontiers in Marine Science, 9, 1–16.35450130

[jfb70044-bib-0024] Kajiura, S. M. , Sebastian, A. P. , & Tricas, T. C. (2000). Dermal bite wounds as indicators of reproductive seasonality and behaviour in the Atlantic stingray, *Dasyatis sabina* . Environmental Biology of Fishes, 58, 23–31.

[jfb70044-bib-0025] Kinney, M. J. , & Simpfendorfer, C. A. (2009). Reassessing the value of nursery areas to shark conservation and management. Conservation Letters, 2, 53–60.

[jfb70044-bib-0026] Knip, D. M. , Heupel, M. R. , & Simpfendorfer, C. A. (2012). Evaluating marine protected areas for the conservation of tropical coastal sharks. Biological Conservation, 148, 200–209.

[jfb70044-bib-0027] Kobori, H. , Dickinson, J. L. , Washitani, I. , Sakurai, R. , Amano, T. , Komatsu, N. , Kitamura, W. , Takagawa, S. , Koyama, K. , Ogawara, T. , & Miller‐Rushing, A. J. (2016). Citizen science: A new approach to advance ecology, education, and conservation. Ecological Research, 31, 1–19.

[jfb70044-bib-0028] Kyne, P. M. , Courtney, A. J. , & Bennett, M. B. (2008). Aspects of reproduction and diet of the Australian endemic skate *Dipturus polyommata* (Ogilby) (Elasmobranchii: Rajidae), by‐catch of a commercial prawn trawl fishery. Journal of Fish Biology, 72, 61–77.

[jfb70044-bib-0029] Kynoch, R. J. , Fryer, R. J. , & Neat, F. C. (2015). A simple technical measure to reduce bycatch and discard of skates and sharks in mixed‐species bottom‐trawl fisheries. ICES Journal of Marine Science, 72, 1861–1868.

[jfb70044-bib-0030] Licandeo, R. , Cerna, F. , & Céspedes, R. (2007). Age, growth, and reproduction of the roughskin skate, *Dipturus trachyderma*, from the southeastern Pacific. ICES Journal of Marine Science, 64, 141–148.

[jfb70044-bib-0031] Licandeo, R. , & Cerna, F. T. (2007). Geographic variation in life‐history traits of the endemic kite skate *Dipturus chilensis* (Batoidea: Rajidae), along its distribution in the fjords and channels of southern Chile. Journal of Fish Biology, 71(2), 421–440.

[jfb70044-bib-0032] Luer, C. A. , & Gilbert, P. W. (1985). Mating behavior, egg deposition, incubation period, and hatching in the clearnose skate, *Raja eglanteria* . Environmental Biology of Fishes, 13, 161–171.

[jfb70044-bib-0033] Luer, C. A. , Walsh, C. J. , Bodine, A. B. , & Wyffels, J. T. (2007). Normal embryonic development in the clearnose skate, *Raja eglanteria*, with experimental observations on artificial insemination. Environmental Biology of Fishes, 80, 239–255.

[jfb70044-bib-0034] Marshall, A. D. , & Bennett, M. B. (2010). Reproductive ecology of the reef manta ray *Manta alfredi* in southern Mozambique. Journal of Fish Biology, 77, 169–190. 20646146 10.1111/j.1095-8649.2010.02669.x

[jfb70044-bib-0035] Marshall, A. D. , & Pierce, S. J. (2012). The use and abuse of photographic identification in sharks and rays. Journal of Fish Biology, 80, 1361–1379. 22497388 10.1111/j.1095-8649.2012.03244.x

[jfb70044-bib-0036] McCallister, M. , Mandelman, J. , Bonfil, R. , Danylchuk, A. , Sales, M. , & Ajemian, M. (2020). First observation of mating behavior in three species of pelagic myliobatiform rays in the wild. Environmental Biology of Fishes, 103, 163–173.

[jfb70044-bib-0037] McInturf, A. G. A. , Bowman, J. , Schulte, J. M. , Newton, K. C. , Vigil, B. , Honig, M. , Pelletier, S. , Cox, N. , Lester, O. , Cantor, M. , & Chapple, T. K. (2023). A unified paradigm for defining elasmobranch aggregations. ICES Journal of Marine Science, 80, 1551–1566.

[jfb70044-bib-0038] McKinley, D. C. , Miller‐Rushing, A. J. , Ballard, H. L. , Bonney, R. , Brown, H. , Cook‐Patton, S. C. , Evans, D. M. , French, R. A. , Parrish, J. K. , Phillips, T. B. , Ryan, S. F. , Shanley, L. A. , Shirk, J. L. , Stepenuck, K. F. , Weltzin, J. F. , Wiggins, A. , Boyle, O. D. , Briggs, R. D. , Chapin, S. F. , … Soukup, M. A. (2017). Citizen science can improve conservation science, natural resource management, and environmental protection. Biological Conservation, 208, 15–28.

[jfb70044-bib-0039] Nozu, R. , Murakumo, K. , Yano, N. , Furuyama, R. , Matsumoto, R. , Yanagisawa, M. , & Sato, K. (2018). Changes in sex steroid hormone levels reflect the reproductive status of captive female zebra sharks (*Stegostoma fasciatum*). General and Comparative Endocrinology, 265, 174–179. 29510152 10.1016/j.ygcen.2018.03.006

[jfb70044-bib-0040] Parent, S. , Pépin, S. , Genet, J. P. , Misserey, L. , & Rojas, S. (2008). Captive breeding of the barndoor skate (*Dipturus laevis*) at the Montreal biodome, with comparison notes on two other captive‐bred skate species. Zoo Biology, 27, 145–153. 19360612 10.1002/zoo.20170

[jfb70044-bib-0041] Piercy, A. , Gelsleichter, J. , & Snelson, F. F. (2006). Morphological changes in the clasper gland of the Atlantic stingray, *Dasyatis sabina*, associated with the seasonal reproductive cycle. Journal of Morphology, 267, 109–114. 16270309 10.1002/jmor.10389

[jfb70044-bib-0042] Pratt, H. L. , & Carrier, J. C. (2001). A review of elasmobranch reproductive behavior with a case study on the nurse shark, *Ginglymostoma cirratum* . Environmental Biology of Fishes, 60, 157–188.

[jfb70044-bib-0043] Pratt, H. L. , Pratt, T. C. , Knotek, R. J. , Carrier, J. C. , & Whitney, N. M. (2022). Long‐term use of a shark breeding ground: Three decades of mating site fidelity in the nurse shark, *Ginglymostoma cirratum* . PLoS One, 17, 1–23. 10.1371/journal.pone.0275323 PMC9576040 36251734

[jfb70044-bib-0044] R Core Team . (2024). R: A language and environment for statistical computing.

[jfb70044-bib-0045] Régnier, T. , Dodd, J. , Benjamins, S. , Gibb, F. M. , & Wright, P. J. (2024). Spatial management measures benefit the critically endangered flapper skate, *Dipturus intermedius* . Aquatic Conservation: Marine and Freshwater Ecosystems, 34, 1–12.

[jfb70044-bib-0046] Séguigne, C. , Mourier, J. , Clua, É. , Buray, N. , & Planes, S. (2023). Citizen science provides valuable data to evaluate elasmobranch diversity and trends throughout the French Polynesia's shark sanctuary. PLoS One, 18, 1–23. 10.1371/journal.pone.0282837 PMC10032523 36947523

[jfb70044-bib-0047] Shibuya, A. , & Duncan, W. P. (2022). Pre‐copulatory bite wounds as evidence of aggressive competition for mating in the neotropical freshwater stingray *Potamotrygon motoro* . Acta Amaz, 52, 45–48.

[jfb70044-bib-0048] Sulikowski, J. A. , Tsang, P. C. W. , & Huntting Howell, W. (2004). An annual cycle of steroid hormone concentrations and gonad development in the winter skate, *Leucoraja ocellata*, from the western gulf of Maine. Marine Biology, 144, 845–853.

[jfb70044-bib-0049] Sutcliffe, R. (1994). Twenty years of tagging common skate and tope off the west coast of Scotland. In Proceedings of the Second European Shark and Ray Workshop (pp. 14–16). Natural History Museum.

[jfb70044-bib-0050] Sutherland, C. , Hare, D. , Johnson, P. J. , Linden, D. W. , Montgomery, R. A. , & Droge, E. (2023). Practical advice on variable selection and reporting using Akaike information criterion. Proceedings of the Royal Society B: Biological Sciences, 290, 20231261. 10.1098/rspb.2023.1261 PMC10523071 37752836

[jfb70044-bib-0051] Talwar, B. S. , Bond, M. E. , Williams, S. , Brooks, E. J. , Chapman, D. D. , Howey, L. A. , Knotek, R. , & Gelsleichter, J. (2023). Reproductive timing and putative mating behavior of the oceanic whitetip shark *Carcharhinus longimanus* in the eastern Bahamas. Endangered Species Research, 50, 181–194.

[jfb70044-bib-0052] Thorburn, J. , Cole, G. , Naylor, A. , Garbett, A. , Wilson, K. , James, M. , Dodd, J. , Houghton, J. D. R. , & Collins, P. C. (2023). Preliminary insight into the reproductive traits of the flapper skate (*Dipturus intermedius*) using in‐field ultrasonography and circulating hormone concentrations. Endangered Species Research, 50, 97–111.

[jfb70044-bib-0053] Thorburn, J. , Wright, P. J. , Lavender, E. , Dodd, J. , Neat, F. , Martin, J. G. A. , Lynam, C. , & James, M. (2021). Seasonal and ontogenetic variation in depth use by a critically endangered benthic elasmobranch and its implications for spatial management. Frontiers in Marine Science, 8, 1–15.35685121

[jfb70044-bib-0054] Van Vliet, K. , & Moore, C. (2016). Citizen Science initiatives: Engaging the public and demystifying Science. Journal of Microbiology & Biology Education, 17, 13–16. 27047582 10.1128/jmbe.v17i1.1019 PMC4798796

[jfb70044-bib-0055] Whitney, N. M. , Pyle, R. L. , Holland, K. N. , & Barcz, J. T. (2012). Movements, reproductive seasonality, and fisheries interactions in the whitetip reef shark (*Triaenodon obesus*) from community‐contributed photographs. Environmental Biology of Fishes, 93, 121–136.

[jfb70044-bib-0056] Womersley, F. , Hancock, J. , Perry, C. T. , & Rowat, D. (2021). Wound‐healing capabilities of whale sharks (*Rhincodon typus*) and implications for conservation management. Conservation Physiology, 9, 1–16. 10.1093/conphys/coaa120 PMC7859907 33569175

[jfb70044-bib-0057] Wood, S. N. (2017). Generalized additive models: An Introduction with R (2nd ed.). Chapman & Hall.

