# Supplementary material for: Using citizen science photographs to identify reproductive events in an oviparous elasmobranch
Source: J Fish Biol. 2025 Apr 8;107(2):419–30. doi: 10.1111/jfb.70044 (PMC12360143; doi:10.1111/jfb.70044)
Supplement: Supplementary file 1 — Data S1. Supporting Information. [file JFB-107-419-s001.pdf]

Supplements for “Using citizen science photographs to identify reproductive events in an oviparous elasmobranch”

Criteria for forming image pools

Pelvic swelling

Poor quality image if:

i. Extreme lighting (shade or glare) across pelvic disc reducing visibility
ii. Poor resolution image with lack of contrast such that it is hard to define features on the skate
iii. Majority of pelvic disc out of view due to obstructions, camera angle etc.

Bite wounds

Poor quality image if:

i. Extreme (e.g. intense shade or glare) or poor lighting
ii. Low resolution or out of focus
iii. Majority of skate out of view, due to obstructions, camera angle etc.
iv. Edges of pectoral fins not visible

Scratch wounds

Poor quality image if:

i. Extreme (e.g. intense shade or glare) or poor lighting over pectoral fins affecting visibility
ii. Low resolution or out of focus
iii. Majority of skate out of view, due to obstructions, camera angle etc.

Criteria for scoring images

Pelvic swelling

Mark “yes” (swollen) if:

i. Swollen appearance, particularly in latter half of pelvic disc, giving pelvic disc an inflated appearance
ii. Lack of definition at edges of back-bone

Examples in Figure S1.

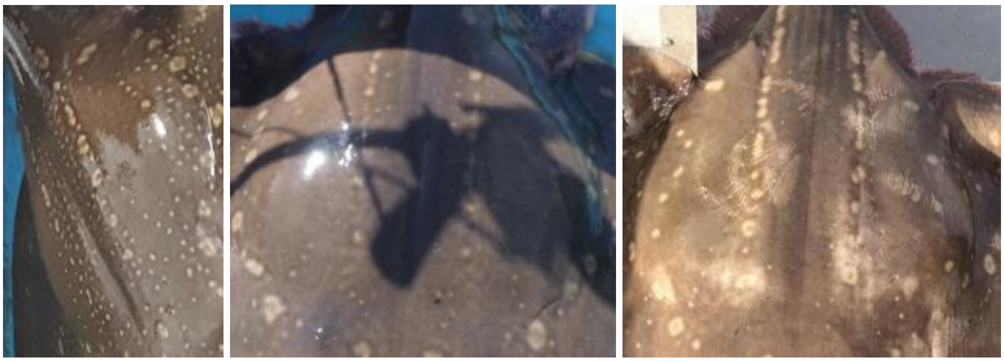

Figure S1. Examples of pelvic swelling

Mark “no” (not swollen) if:

i. Pronounced depression in central disc
ii. No swelling or bulging

Examples in Figure S2.

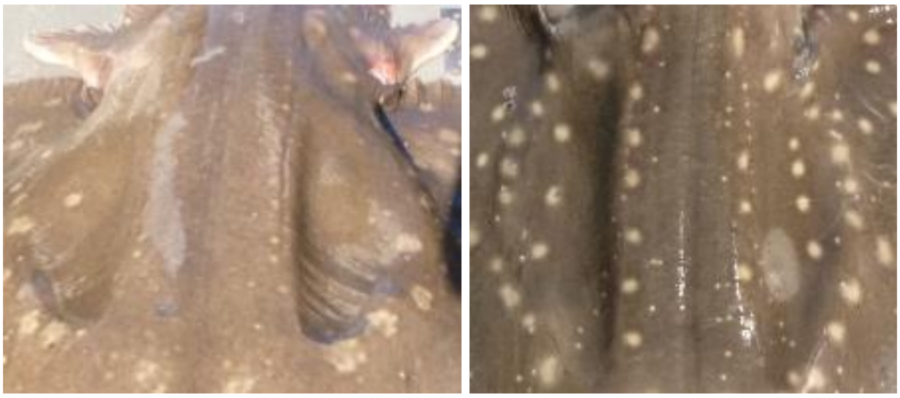

Figure S2. Example of no pelvic swelling.

Mark “undetermined” if:

i. Lack of major swelling coupled with no major depression
ii. Back-bone defined along the edge without general pronounced depression

Example in Figure S3.

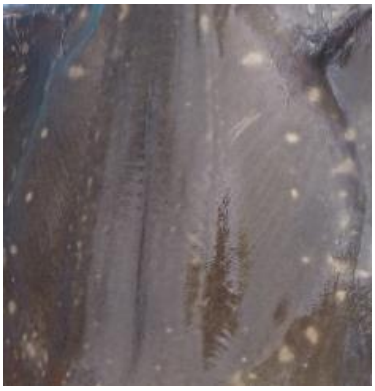

Figure S3. Example of a skate where the status of pelvic swelling is undetermined.

Bite wound

Record as positive occurrence of bite wound if:

i. U-shaped collection of equidistant marks, generally around edge of body. Example in Figure S4.
ii. Triangular arrangement of scratch equidistant marks on wings or pelvic disc
iii. Single-track or double-track equidistant scrapes on body surface. Example in Figure S5.
iv. Bite(s) not visible in previous image of individual (if applicable)

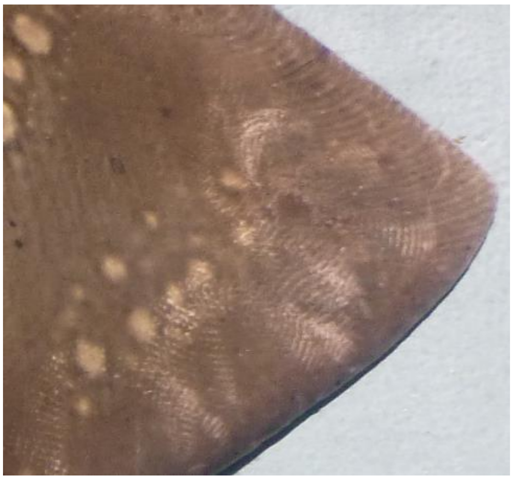

Figure S4. Example of U-shaped bite wounds.

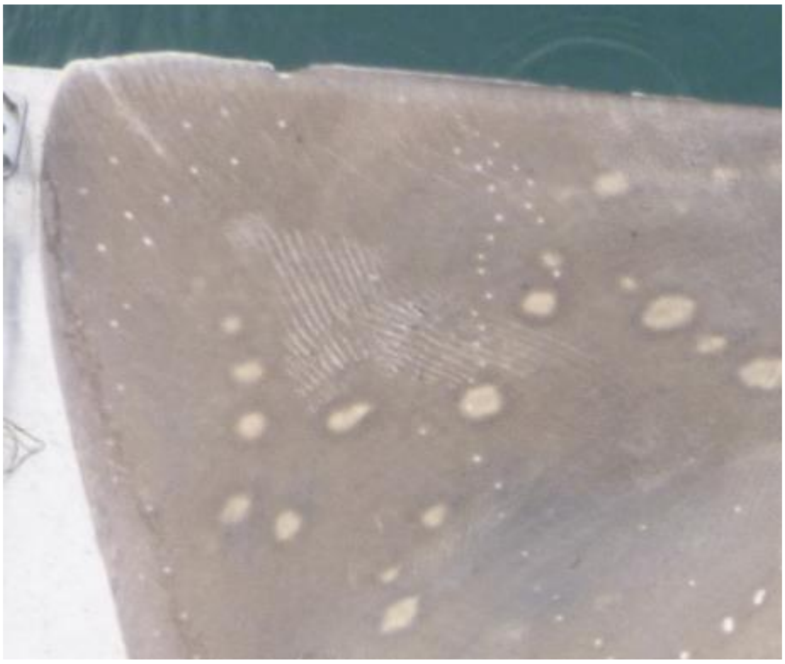

Figure S5. Example of track-like bite wounds.

Separately record bites which are new to the observer and general presence e.g. record new bites and overall presence.

Scratch wound

Record as scratch wound if:

i. Marks running approximately parallel to the anterior-posterior axis across the pectoral fin(s)
ii. Marks are not a clear, distinct, constant line
iii. Scratch(es) not visible in previous image of individual (if applicable/possible to determine)

Example in Figure S6.

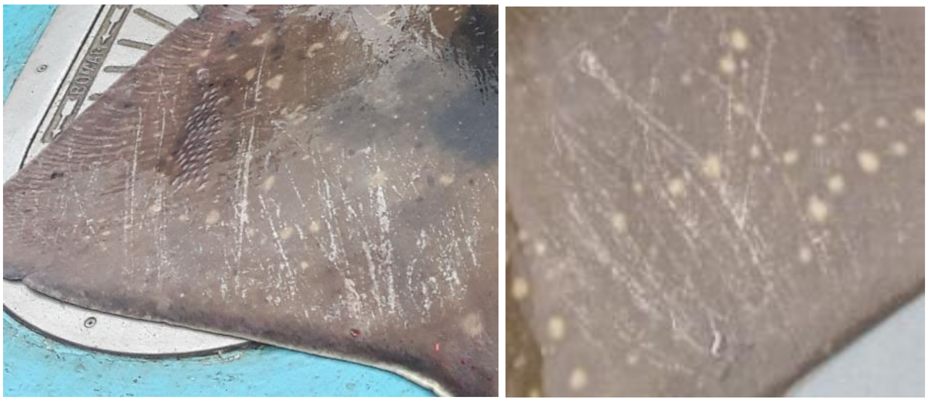

Figure S6. Example of scratch wounds.

An example of what not to consider as a scratch wound is shown in Figure S7.

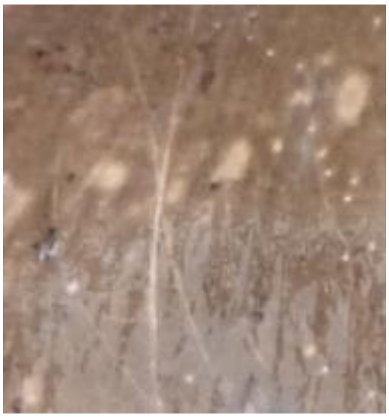

Figure S7. Example of scratch-like wounds not considered in this study.

Model selection table

Table S1. Model selection process. The table shows the backwards stepwise model selection process used to determine which variables affected on the abundance of bite wounds, scratch wounds and pelvic swelling. The table shows the degrees of freedom and Akaike's Information Criterion (AIC) associated with each model. The best model is shown in bold and the ΔAIC column shows the difference in AIC between a model and the final model. Only fixed effect terms are show – models also included skipper and year as random variables

Response Variable	Model	Model terms	Degrees of freedom	AIC	ΔAIC
Bite wounds	Saturated	sex + region + sex:region + s(day, bs='cc', by=sex)+ s(day, bs='cc', by=region)	15.83	741.12	8.00
	1	sex+region+sex:region +s(day, bs='cc', by=region)	10.34	736.25	3.13
	2	sex+region+sex:region + s(day, bs='cc')	10.16	734.20	1.08
	3	sex + region + s(day, bs='cc')	8.93	734.45	1.33
	**4**	**sex + s(day, bs='cc')**	**8.48**	**733.12**	**0**
	5	sex	7.50	737.82	4.7
	6	s(day, bs='cc')	5.87	762.45	29.33
Scratch wounds	Saturated	sex+region+sex:region+ s(day, bs='cc', by=region)+ s(day, bs='cc', by=sex)	17.42	390.35	6.37
	1	sex+region+sex:region+ s(day, bs='cc', by=region)	13.33	388.92	4.94
	2	sex+region+sex:region + s(day, bs='cc')	10.81	385.51	1.53
	3	sex+region +s(day, bs='cc')	9.42	384.79	0.99
	**4**	**sex + s(day, bs='cc')**	**9.45**	**383.98**	**0**
	5	sex	14.58	546.92	162.94
	6	s(day, bs='cc')	8.97	440.65	56.67
Pelvic swelling	Saturated	region + s(day, bs='cc', by=region)	22.13	828.53	5.58
	1	region + s(day, bs='cc')	15.48	824.62	1.67
	**2**	**s(day, bs='cc')**	**15.09**	**822.95**	**0**
	3	1	9.59	862.98	40.03
